# Improvement of rooting and growth in kiwifruit (*Actinidia deliciosa*) cuttings with organic biostimulants

**DOI:** 10.1016/j.heliyon.2023.e17815

**Published:** 2023-06-30

**Authors:** Sudip Kumar Dutta, Jayanta Layek, Ashish Yadav, Shaon Kumar Das, Heiplanmi Rymbai, Somnath Mandal, Nandita Sahana, T.L. Bhutia, E.L. Devi, V.B. Patel, Ramgopal Laha, V.K. Mishra

**Affiliations:** a ICAR Research Complex for NEH Region, Sikkim Centre, Gangtok, Sikkim, 737 102, India; b ICAR Research Complex for NEH Region, Umiam, Meghalaya, 793 103, India; c Department of Biochemistry, Uttar Banga Krishi Viswavidyalaya, Pundibari, 736165, Cooch Behar, West Bengal, India; d Horticultural Science Division, Indian Council of Agricultural Research, KAB II, New Delhi, 110012, India

**Keywords:** Kiwifruit, Seaweed, Humic acid, Cutting, Propagation, Rooting, Growth, *Actinidia deliciosa*

## Abstract

Seaweed extracts have shown profoundly positive effects on crop growth, quality and reproduction in diverse agricultural and horticultural crops. Seaweed extracts can be used to promote the rooting and growth of cuttings in perennial fruit species like kiwifruit (*Actinidia deliciosa*). In this study, the cuttings were treated with 1, 5, 10 and 50% solutions of G Sap (*Gracilaria edulis*), K Sap (*Kappaphycus alvarezii*), AN (*Ascophyllum nodosum*), EM (*Ecklonia maxima*), HA (Humic acid) and control (water) for 6 h as base dipping. Subsequently, the treatments of G Sap, K Sap, AN, EM, HA and control were repeated every 15 days for a period of six months as application of 50 ml solutions in the potted cuttings. All the treatments exhibited significant effects on the rooting percent in all the kiwifruit cultivars, namely ‘Monty’, ‘Abott’, ‘Hayward’, ‘Allison’ and ‘Bruno’ (*P* ≤ 0.01) as compared to the control. Shoot and root growth parameters including leaf number per cutting, number of roots per cutting, number of branches, plant height, shoot diameter, root length, root diameter and root weight were all positively increased with the application of seaweed extracts (*P* ≤ 0.05). Cuttings treated with seaweed extract exhibited significantly higher levels of pigments (chlorophyll *a*, chlorophyll *b* and total carotenoids), metabolites (total carbohydrates and soluble phenols) and less electrolyte leakage as compared to the control cuttings. Significant positive and negative correlations were observed between biochemical parameters combined with plant nutrient concentration. Principal component analysis (PCA) revealed that PC1 and PC2 (first two principal components) accounted for 75% of the entire variation. While, PC1 accounted for 63% of the total variation, PC2 accounted for 11% of the total variation. The leaves and the roots of kiwifruit cultivar ‘Hayward’ treated with G Sap at 10%, K Sap at 10%, AN at 10%, EM at 10%, HA at 10% exhibited higher expression of all four root promoting candidate genes (*GH3-3, LBD16, LBD29* and *LRP1*) compared to the control. Therefore, it can be concluded that, seaweed extract and humic acid can be used as a suitable alternative to synthetic hormones for promoting the rooting and growth of kiwifruit cuttings.

## 1 Introduction

Kiwifruit, botanically known as *Actinidia chinensis* Planch. and *A. deliciosa* (A. Chev.) C.F.Liang et A.R. Ferguson are the two major closely related species grown worldwide [1]. Among these two, *A. deliciosa* is the predominant species of kiwifruit grown worldwide and requires cooler and wetter conditions, whereas *A. chinensis* is adapted to the warmer altitudes of eastern China [1]. As per the Food and Agriculture Organisation (FAO) of the United Nations, globally, 4,348,011 metric tonnes of kiwifruit are produced from an area of 268,788 ha; and China is the leading producer of kiwifruit, followed by Italy, New Zealand, Chile and Greece who are the major producer countries with a total world export value of 2,907,580 thousand US dollars (http://www.fao.org/faostat/en/#data/QC). The fruits of kiwifruit are not only nutritionally rich but also enhance immunity, digestion and metabolism; their health benefits are well documented in the literature [2,3]. Kiwifruit is a significantly important source of vitamin C, vitamin E, dietary fibre, potassium, antioxidants, folate and several enzymes [2]. Actinidin is a proteolytic enzyme present exclusively in kiwifruit that has been found to have a positive effect on gastric protein digestion and is known for psychological well being [[4], [5], [6]].

The most popular varieties of kiwifruit in the world include Huayou, Hort 16A, Hongyang, Qinmei, Xuxiang, Yate, Haywai and Kuilv (China), Abbot, Bruno, Elmwood, Gracie and Monty (Italy), Abbott, Monty, Bruno, and Hayward (New Zealand) [7,8]. ‘Hayward’ remains the single most dominant variety of the world kiwifruit industry owing to its popularity with consumers and growers due to its taste, size and storage qualities [8]. It’s a well known fact that organic fruits in general are largely preferred by consumers worldwide due to their safety and health benefits. In a study in north-western Spain, cultivar ‘Hayward’ was evaluated under organic, inorganic and integrated farming systems (IFS) for physicochemical and sensory attributes. While, the organic Kiwis were found to contain more citric acid than the inorganic and IFS systems, vitamin C and total phenol were recorded at par in all three systems, sweetness and juiciness were also on par with the conventional system [9]. Several other studies found the possibility of enhancement of fruit quality and nutrient concentration with the use of various bio-organic inputs, resolved myth versus reality and explored the pharmaceutical properties of organically grown kiwifruit [[10], [11], [12]].

Although organic kiwifruit production is an alternate production system in many developed countries, it is still constrained by a lot of operational challenges like the limited availability of organic nutrient inputs, organic growth regulators, pesticides etc. Among these factors, the absence of an organically permitted growth regulator is one of the major issues. Kiwifruit is generally clonally propagated through hardwood cutting and the success of rooting of cutting depends heavily on the use of synthetic auxins which is not permitted in organic farming. Seed propagation is slow and the hybrid progeny is not true to the type like the parents. During our preliminary studies we found very less rooting (<10%) in hardwood kiwifruit cuttings. In the case of organic farming, it is very important to get fast rooting, a high rooting percentage and improved survival of cuttings for commercial propagation of kiwifruit.

There are many studies having positive results with various inorganic treatments in softwood cuttings [[13], [14], [15], [16]] and also nutrient media in micro-propagation techniques [[17], [18], [19], [20]]. Seaweeds are marine brown, red and green microalgae, that are commonly utilised in horticultural crops for a variety of reasons, including their ability to promote plant development and improve crop tolerance to abiotic stresses, such as severe temperatures, nutrient deficiencies, drought, etc. Moreover, humic acids are organic compounds formed by the decay of plants and animals. However, seaweed sap and humic acid are found to be potential sources of phytohormones (auxin, cytokinin, gibberalic acid, abscissic acid and polyamines) which have been established in several studies [[21], [22], [23], [24], [25], [26], [27]]. This implies that using seaweed extract and humic acid might increase the likelihood of kiwifruit that will successfully root, making it a useful tool for growing kiwifruit plants under an organic production system. However, it's important to note that, more research is needed to completely comprehend the impact of seaweed extract and humic acid on kiwifruit rooting and how to optimally use them in different growing conditions. The effects of these organic inputs on rooting behavior and their impact on biochemical and nutritional parameters of Kiwifruit cuttings are largely unknown. Whether, these inputs have any upregulatory effect on rooting-related genes at the molecular level is still a question to be answered. It was hypothesised that these two classes of organic compounds can act as a suitable alternative to synthetic auxins in the propagation of cuttings. With this background, the aims of these experiments were to evaluate the effect of seaweed extract and humic acid on rooting and growth in kiwifruit cuttings. More specifically, we looked into the effects of seaweed sap and humic acid on biochemical and nutrient parameters and also on root-inducing genes (*GH3-3, LBD16, LBD29* and *LRP1*) in ‘Hayward’ cultivar of kiwifruit.

## 2 Materials and methods

### 2.1 Plant material and rooting conditions

Kiwifruit is a dioecious, climber shrub of the semi-woody type and takes 3–4 years to start flowering and fruiting if propagated clonally by hardwood cuttings. Cuttings were selected from female plants (10–12 years old) of the kiwifruit cultivars ‘Monty’, ‘Abott’, ‘Hayward’, ‘Allison’ and ‘Bruno’. Cuttings of 8–10 mm diameter and 30 cm long were selected from healthy and actively growing plants during the month of January 2020 (dormant season). The cuttings were collected from the experimental farm (kiwifruit block) of ICAR RC NEH Region, Sikkim Centre, Gangtok, Sikkim, India (27 19 11.339 N, 88 36 9.793 E and 1348 m AMSL). All the experiments were conducted in a polyhouse at the same location. LDPE polybags of dimensions 5 × 7 Inches were used for placing the cuttings; and one cutting was planted in each of the polybags. Cuttings were planted into a rooting media consisting of vermiculite, perlite and cocopeat in a ratio of 1:1:1 [28]. Irrigation was done using a microsprinkler irrigation system (with a capacity of 30 L h^−1^m^−2^) for 10 min per day. At the ICAR RC NEH Region, Sikkim Centre, Gangtok, Sikkim, India, plants were cultivated on benches in a glasshouse with environment control (heating below 15 °C and cooling over 27 °C).

### 2.2 Treatment details

Immediately after harvesting the cuttings, the cuttings were treated with 1, 5, 10 and 50% solutions of G Sap (*Gracilaria edulis*), K Sap (*Kappaphycus alvarezii*), AN (*Ascophyllum nodosum*), EM (*Ecklonia maxima*), HA (humic acid) and control for 6 h by dipping the base of the cuttings in a plastic bucket. Out of five treatments, we have used four seaweed extracts (G Sap, K Sap, AN and EM) and one Humic acid (HA); all were organic treatments. The nutrient and other compositions of the treatments are given in Supplemental Table A. After these treatments, the cuttings were transferred into LDPE polybags for rooting in the polyhouse. The treatments of 1, 5, 10 and 50% solutions of G Sap, K Sap, AN, EM, HA and control (only water) were repeated by putting 50 ml of solutions in the rooting zone of the cuttings every 15 days for a period of six months. Thereafter, all the growth and other parameters were measured after six months of planting.

### 2.3 Shoot and root growth parameters

Rooted cuttings (%) were counted out of the total cuttings. Leaf number, number of roots per cutting and number of branches were counted manually. Plant height and root length were measured with a measuring scale and expressed in centimetres. Shoot diameter, root diameter were measured with a digital calliper (SDN20, BAKER®) and expressed in millimetres. The root weight of the cutting was measured with an electronic balance (Aczel®).

### 2.4 Biochemical parameters

Chlorophyll *a*, Chlorophyll *b*, total chlorophyll and total carotenoid content were measured using a standardised method with some modifications [[29], [30], [31]] and expressed as mg/g fresh weight (fw). Mature leaves (10 mg) were immersed in 10 ml of dimethyl sulphoxide (DMSO) and incubated at 70 °C (in a hot air oven) for 4 h. After that, the samples were read on a spectrophotometer (Biospectrometer, Eppendorf, Germany) at 645, 663 and 480 nm (with DMSO as a blank).

Chlorophyll *a*, Chlorophyll *b* and total carotenoids content were calculated using the following formula:

Chlorophyll *a* (mg g^−1^ fw) = (12.7 × OD_663_) − (2.69 × OD_645_) × Volume × dilution/1000 × weight of sample

Chlorophyll *b* (mg g^−1^ fw) = (22.7 × OD_645_) − (4.68 × OD_645_) × Volume × dilution/1000 × weight of sample

Total Chlorophyll (mg g^−1^ fw) = Chlorophyll *a* + Chlorophyll *b*

Total carotenoids (mg g^−1^ fw) = 1000 A_470_ − (3.27 Chl-a + 1Chl-b)/229

Anthrone reagent method was used for estimation of total carbohydrate content [32]. Fresh leaf (200 mg) was boiled for 3 h in 3 mL 2.5 N HCl and then neutralised with sodium carbonate. Then volume was made up to 5 mL with double distilled water and centrifuged at 3000 *g* for 15 min at 25 °C. Volume was made up to 1 ml (100 μL supernatant + double distilled water) and 4 mL of anthrone reagent was added. The mixture was heated for 8 min and absorbance was recorded at 630 nm. Total carbohydrate content was calculated using a standard curve of 0–100 μg glucose and expressed as μg/g fw.

Electrolyte leakage was measured using a standard procedure with the following formula and was expressed in percentage [33].

Electrolyte leakage (%) = (ECb − ECa)/ECc × 100 where ECa: initial EC, ECb: EC after incubation at 55 °C for 25 min and ECc: after boiling at 100 °C for 10 min.

For estimation of total phenol, the Folin-Ciocalteau reagent and Na_2_CO_3_ (sodium carbonate) solution method were used. Using distilled water, 200 μL of crude extract (1 mg/mL) were diluted to 3 mL, mixed well with 0.5 mL of Folin-Ciocalteu reagent for 3 min, and then added to 2 mL of 20% (w/v) sodium carbonate. The combination was left to stand for a further 60 min in the dark, after which the absorbance at 650 nm was determined. The total phenol content was expressed as mg gallic acid equivalent g^−1^fw [34].

### 2.5 Plant nutrient concentration

Plant samples were prepared by mixing equal quantities of root and shoot, oven drying at 65 °C for 72 h and powdering and sieving with 0.5 mm mesh. The Micro-Kjeldahl method was used for the estimation of total nitrogen (N) [35]. Phosphorus (P) content was determined using an ammonium molybdate assay wherein plant tissues were digested in HNO_3_ and HClO_4_ [36]. Potassium (K) was determined using a flame photometer [37]. Ca (%), Mg (%), were estimated by atomic absorption spectrophotometry (Analyst 200 Atomic Absorption Spectrometer, PerkinElmer) [38] and total sulphur (%) was determined by flame emission photometry and the turbidimetric method, respectively, in the di-acid extract [39].

### 2.6 Gene expression

TRIzol reagent (Invitrogen, Carlsbad, CA) and the RNA MiniPrep kit (Zymo Research, Irvine, CA) were used to extract total RNA from kiwifruit cutting leaves and roots according to the recommended technique [40]. We chose five genes, viz. *GH3-3, LBD16, LBD29 and LRP1* from the RNA-seq data of *Arabidopsis thaliana* L. (Brassicaceae) to validate the expression of genes by treatment with G Sap at 10%, K Sap at 10%, AN at 10%, EM at 10%, HA at 10% and control [41]. Primers having an amplification efficiency of more than 1.97 were used for qPCR. Supplemental Table B contains the primers that were designed using Primer Express v3.0.1. Three biological replications were used in RT-qPCR which was done in the QuantStudio 3 Real-Time PCR System (Applied Biosystems, Foster City, CA, USA) using FastStart SYBR Green (Roche). The expression was normalised with the *UBC21* gene and mean relative gene expression was calculated by the 2^−ΔΔCt^ method [42].

### 2.7 Statistical analysis

The mean values were derived for different parameters using three replications for each treatment. The analysis of variance (ANOVA) approach was used to examine the data for the completely randomised design (CRD). For comparing the treatment means, the least significant difference (LSD) at the 5% level of significance (P = 0.05) was determined, and non-significant treatments were denoted as NS. The software SPSS-17 was used to analyse the data [43]. The R programme was utilised to do Principal Component Analysis (PCA) [44].

## 3 Results

### 3.1 Rooted cutting (%)

**Fig. 1:**
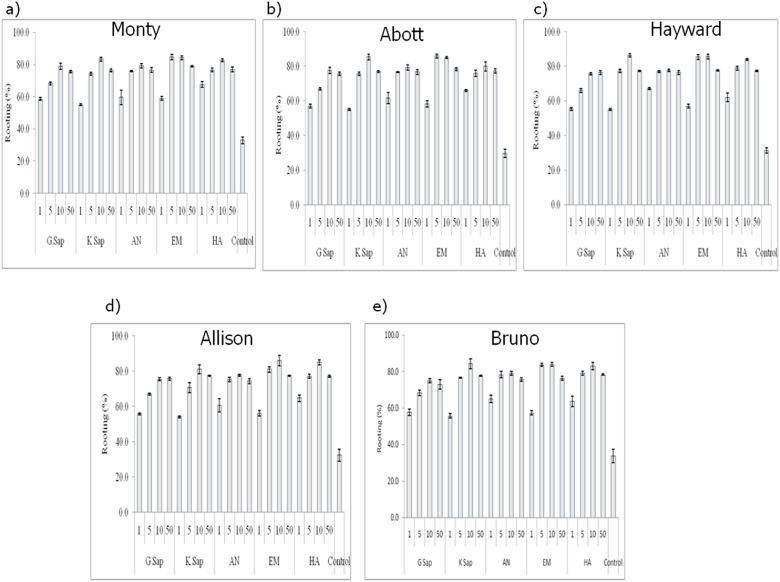
Effect of various treatments (1, 5, 10 and 50% solutions of G Sap, K Sap, AN, EM, HA and control) on rooting of kiwifruit cultivars ‘Monty’, ‘Abott’, ‘Hayward’, ‘Allison’ and ‘Bruno’. The treatment effect exhibited the following F values and level of significance: a) Monty, F- value = 63.09** b) Abott, F- value = 85.63** c) Hayward, F- value = 151.48** d) Allison, F- value = 58.88** e) Bruno, F- value = 48.18** (** indicates statistically significant differences at *P* ≤ 0.01).

Fig. 1(a–e) shows the effect of various treatments (1, 5, 10 and 50% solutions of G Sap, K Sap, AN, EM, HA and control) on the rooting of four Kiwifruit cultivars *viz*. ‘Monty’, ‘Abott’, ‘Hayward’, ‘Allison’ and ‘Bruno’. All the treatments exhibited a significant effect on the rooting percent in all the kiwifruit cultivars (*P* ≤ 0.01). In the cultivar Monty, we observed a 2.56 fold difference (highest with EM at 5% and lowest in control) in the rooting with the treatments. Similarly, in the cultivar Abott, the highest rooting was found in EM at 5% (86.0%) and the lowest in control (29.66%) (2.89 fold difference). In the cultivar Hayward, the highest rooting was found with K Sap at 10% (86.33%) and the lowest in control (31.33%) (2.75 fold difference). In the cultivar Allison, the highest rooting was found in EM at 10% (86.00%) and lowest in control (32.33%) (2.66 fold difference). In the cultivar Bruno, we observed a 2.50 fold difference (highest with EM at 10% and lowest in control) in the rooting with the treatments. It was observed that 50% solutions of G Sap, K Sap, AN, EM and HA did not give any significant increase or highest rooting in any of the cultivars, therefore, this treatment was not taken forward for further analysis (Fig. 1).

### 3.2 Shoot and root growth parameters

**Fig. 2:**
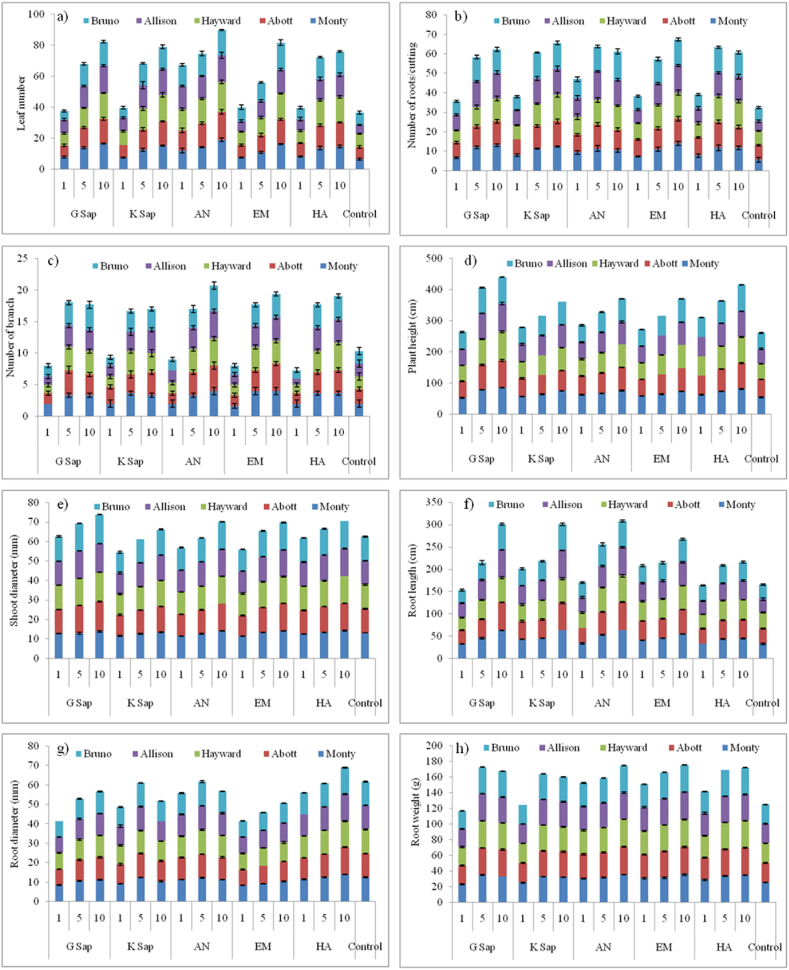
Effect of various treatments (1, 5 and 10% solutions of G Sap, K Sap, AN, EM, HA and control) on shoot and root growth parameters of kiwifruit cultivars ‘Monty’, ‘Abott’, ‘Hayward’, ‘Allison’ and ‘Bruno’.

The effects of various treatments (1, 5 and 10% solutions of G Sap, K Sap, AN, EM, HA and control) on shoot and root growth parameters of Kiwifruit cultivars ‘Monty’, ‘Abott’, ‘Hayward’, ‘Allison’ and ‘Bruno’ are represented in Fig. 2. Leaf number per cutting varied significantly (*p* ≤ *0.05*) with the application of treatments (Fig. 2a); the best treatments were AN at 10% (19.00) in Monty, AN at 10% (18.00) in Abott, AN at 10% (19.33) in Hayward, G Sap at 10% (17.66) in Allison and EM at 10% (17.33) in Bruno respectively. The number of roots per cutting exhibited significant (*p* ≤ *0.05*) variation with the application of treatments (Fig. 2b); the treatments that induced maximum effect were EM at 10% (14.00) in Monty, HA at 5% (13.33) in Abott, K Sap at 10% (13.66) in Hayward, AN at 5% (14.66) in Allison and AN at 10% (14.66) in Bruno respectively. The number of branches varied significantly (*p* ≤ *0.05*) with the application of treatments (Fig. 2c); highest number of branches were recorded with AN at 10%, EM at 5 and 10% (4.00) in Monty, EM at 10% (4.33) in Abott, AN and HA at 10% (4.33) in Hayward, AN at 10% (4.33) in Allison and G Sap and AN at 10% (4.00) in Bruno respectively. Plant height exhibited significant (*p* ≤ *0.05*) variation with the application of treatments (Fig. 2d); the treatments that induced the maximum effect were G Sap at 10% (85.00 cm) in Monty, G Sap at 10% (87.33 cm) in Abott, G Sap at 10% (91.00 cm) in Hayward, G Sap at 10% (90.67 cm) in Allison and G Sap at 10% (84.67 cm) in Bruno respectively. Shoot diameter varied significantly (*p* ≤ *0.05*) with the application of treatments (Fig. 2e); highest shoot diameters were recorded with HA at 10% (14.27 mm) in Monty, G Sap at 10% (15.33 mm) in Abott, G Sap at 10% (15.33 mm) in Hayward, G Sap at 10% (14.70 mm) in Allison and G Sap at 10% (14.80 mm) in Bruno respectively. Root length exhibited significant (*p* ≤ *0.05*) variation with the application of treatments (Fig. 2f); the treatments that induced the maximum effect were K Sap at 10% (63.33 cm) in Monty, G Sap at 10% (63.00 cm) in Abott, AN at 10% (59.00 cm) in Hayward, AN at 10% (64.33 cm) in Allison and K Sap at 10% (58.67 cm) in Bruno respectively. Root diameter varied significantly (*p* ≤ *0.05*) with the application of treatments (Fig. 2g); highest root diameters were recorded with HA at 10% (14.03 mm) in Monty, HA at 10% (13.90 mm) in Abott, HA at 10% (13.43 mm) in Hayward, HA at 10% (13.91 mm) in Allison and HA at 10% (13.70) in Bruno respectively. Root weight exhibited significant (*p* ≤ *0.05*) variation with the application of treatments (Fig. 2h); the treatments that induced the maximum effect were AN at 10% (35.43 g) in Monty, AN at 10% (35.53 g) in Abott, AN at 10% (35.11 g) in Hayward, EM at 10% (35.01 g) in Allison and AN at 10% (34.71 g) in Bruno respectively.

### 3.3 Biochemical parameters

In the kiwifruit cultivar ‘Hayward’, significant differences (*p* ≤ *0.05*) were recorded in biochemical parameters after 60 days due to treatment with seaweed extract (Table 1). A 1.95 times variation in chlorophyll *a* concentration in leaves was found between the HA at10% (maximum) and G Sap 1 and 5% (minimum). For chlorophyll *b*, 1.40-fold variation in leaves was observed between the highest (G Sap at10%) and lowest (AN at 5%) treatments, whereas, total chlorophyll content exhibited a 1.44 times variation between the highest (G Sap at 10%) and lowest (G Sap at 1%) treatments. In total carotenoid content, a 1.62 times variation was found between the highest (EM at10%) and lowest (G Sap at1%) treatments. In the case of total carbohydrate content, a 1.80-fold variation was recorded between the maximum (EM at 10%) and minimum (control) treatments. While, electrolyte leakage exhibited a 1.61-fold variation between the control (highest) and AN at 10% (lowest) treatments, whereas, soluble phenols exhibited a 1.21-fold variation between the G Sap at10% (highest) and AN at 5% (lowest) treatments.

**Table 1:** Effect of various treatments (1, 5 and 10% solutions of G Sap, K Sap, AN, EM, HA and control) on biochemical parameters of kiwifruit cultivar ‘Hayward’.

Treatment	Concentration (%)	Chlor a (μg mg^−1^fw)	Chl b (μg mg^−1^fw)	Chlor T (μg mg^−1^fw)	Total carotenoid (mg g^−1^fw)	Total carbohydrate content (μg g^−1^fw)	Electrolyte leakage (%)	Soluble phenols (mg g^−1^fw)
G Sap	1	0.36 ± 0.01	2.61 ± 0.12	2.98 ± 0.12	138.00 ± 2.08	26.67 ± 0.67	20.67 ± 0.88	13.83 ± 0.15
	5	0.36 ± 0.01	2.90 ± 0.02	3.28 ± 0.02	148.00 ± 1.73	28.33 ± 1.33	19.33 ± 0.88	14.40 ± 0.15
	10	0.65 ± 0.02	3.64 ± 0.06	4.29 ± 0.06	171.67 ± 3.71	34.67 ± 0.88	17.33 ± 0.88	15.57 ± 0.20
K Sap	1	0.58 ± 0.01	2.78 ± 0.06	3.36 ± 0.06	178.00 ± 1.15	26.33 ± 0.88	20.60 ± 0.83	12.93 ± 0.26
	5	0.57 ± 0.01	2.76 ± 0.03	3.33 ± 0.02	176.67 ± 1.20	28.00 ± 0.58	17.00 ± 0.58	13.13 ± 0.30
	10	0.65 ± 0.02	2.88 ± 0.06	3.53 ± 0.05	189.33 ± 1.20	35.67 ± 0.88	16.00 ± 0.58	13.63 ± 0.18
AN	1	0.47 ± 0.01	2.87 ± 0.06	3.34 ± 0.06	169.33 ± 1.20	28.67 ± 1.20	19.67 ± 0.67	12.87 ± 0.23
	5	0.57 ± 0.01	2.59 ± 0.03	3.16 ± 0.04	168.00 ± 0.58	30.33 ± 1.33	17.67 ± 0.88	12.83 ± 0.22
	10	0.66 ± 0.01	2.87 ± 0.06	3.53 ± 0.05	187.67 ± 1.76	35.33 ± 0.88	13.67 ± 0.88	13.77 ± 0.23
EM	1	0.47 ± 0.01	2.62 ± 0.09	3.09 ± 0.10	179.67 ± 1.45	29.33 ± 0.88	19.33 ± 0.88	13.67 ± 0.30
	5	0.56 ± 0.01	2.64 ± 0.06	3.20 ± 0.07	215.33 ± 3.48	27.00 ± 0.58	18.00 ± 0.58	14.07 ± 0.23
	10	0.67 ± 0.01	2.91 ± 0.03	3.59 ± 0.03	224.67 ± 4.70	38.00 ± 1.73	16.33 ± 0.88	14.40 ± 0.26
HA	1	0.51 ± 0.01	2.85 ± 0.09	3.35 ± 0.08	138.33 ± 1.76	25.67 ± 0.67	20.00 ± 1.15	13.47 ± 0.26
	5	0.59 ± 0.01	3.35 ± 0.09	3.93 ± 0.10	158.67 ± 2.03	29.00 ± 0.58	18.67 ± 0.88	13.07 ± 0.23
	10	0.70 ± 0.01	3.49 ± 0.03	4.19 ± 0.04	207.67 ± 2.96	32.33 ± 1.76	16.67 ± 0.88	15.13 ± 0.23
Control		0.40 ± 0.03	2.60 ± 0.12	3.00 ± 0.15	155.67 ± 6.57	21.00 ± 1.15	22.00 ± 1.15	13.20 ± 0.29
LSD (*p* < 0.05)		0.03	0.18	0.19	8.22	3.12	2.43	0.30
*F*-value		101.59**	25.90**	36.03**	77.66**	16.35**	6.19**	59.04**

Data are presented as the mean ± SeM (n = 3); LSD, least significant difference; NS, non-significant; **significant at p < 0.01.

### 3.4 Plant nutrient concentration

Among the macronutrients analyzed in the cultivar ‘Hayward’, N and P recorded highly significant variation (*p* ≤ *0.01*), while variation in K was found to be only significant (*p* ≤ *0.05*) (Table 2). In addition, all the micronutrients (Ca, Mg and S) recorded highly significant variation (*p* ≤ *0.01*) (Table 2). Maximum N content was found in K Sap at 5% while the minimum was recorded in control (1.23 fold variation). In the case of P content, a 1.21-fold variation was found between the highest (K Sap at10%) and lowest (control) treatments. K content exhibited relatively less variation (1.13 fold) between the highest (K Sap and HA at 10%) and lowest (HA at 1%) treatments. Ca content varied by 1.31 fold between the highest (AN at 10%) and lowest (K Sap at 1%) treatments. In the case of Mg, a 1.4 fold variation was observed between the highest (EM at 10%) and lowest (AN and K Sap at 1%) treatments. While S content exhibited 1.27 fold variation among the highest (EM at 10%) and lowest (K Sap at 1%) treatments.

**Table 2:** Effect of various treatments (1, 5 and 10% solutions of G Sap, K Sap, AN, EM, HA and control) on nutrient parameters of kiwifruit cultivar ‘Hayward’.

Treatment	Concentration (%)	N (%)	P (%)	K (%)	Ca (%)	Mg (%)	S (%)
G Sap	1	4.10 ± 0.12	0.49 ± 0.01	2.43 ± 0.07	1.83 ± 0.03	0.26 ± 0.01	0.45 ± 0.01
	5	4.40 ± 0.10	0.51 ± 0.01	2.47 ± 0.09	1.97 ± 0.07	0.25 ± 0.00	0.47 ± 0.01
	10	4.53 ± 0.12	0.56 ± 0.01	2.63 ± 0.09	2.03 ± 0.03	0.33 ± 0.01	0.54 ± 0.01
K Sap	1	3.77 ± 0.09	0.51 ± 0.01	2.60 ± 0.12	1.67 ± 0.03	0.25 ± 0.01	0.44 ± 0.01
	5	4.63 ± 0.09	0.53 ± 0.01	2.57 ± 0.09	1.77 ± 0.12	0.26 ± 0.01	0.47 ± 0.01
	10	4.60 ± 0.06	0.57 ± 0.01	2.73 ± 0.09	1.93 ± 0.03	0.34 ± 0.01	0.53 ± 0.01
AN	1	3.77 ± 0.12	0.52 ± 0.01	2.43 ± 0.09	1.67 ± 0.09	0.25 ± 0.00	0.47 ± 0.01
	5	4.33 ± 0.09	0.54 ± 0.01	2.67 ± 0.09	1.93 ± 0.03	0.26 ± 0.01	0.47 ± 0.00
	10	4.60 ± 0.06	0.58 ± 0.01	2.50 ± 0.06	2.10 ± 0.06	0.30 ± 0.00	0.53 ± 0.01
EM	1	3.80 ± 0.06	0.54 ± 0.01	2.53 ± 0.09	1.97 ± 0.09	0.26 ± 0.00	0.46 ± 0.01
	5	4.20 ± 0.06	0.53 ± 0.01	2.70 ± 0.06	1.83 ± 0.13	0.26 ± 0.01	0.47 ± 0.01
	10	4.53 ± 0.03	0.55 ± 0.01	2.67 ± 0.09	2.07 ± 0.09	0.35 ± 0.01	0.56 ± 0.01
HA	1	3.77 ± 0.07	0.50 ± 0.01	2.40 ± 0.06	1.80 ± 0.10	0.27 ± 0.01	0.51 ± 0.01
	5	4.27 ± 0.09	0.54 ± 0.01	2.63 ± 0.09	1.93 ± 0.03	0.27 ± 0.01	0.53 ± 0.01
	10	4.53 ± 0.03	0.56 ± 0.01	2.73 ± 0.07	2.07 ± 0.07	0.34 ± 0.01	0.53 ± 0.01
Control		3.77 ± 0.07	0.48 ± 0.01	2.50 ± 0.15	1.80 ± 0.17	0.27 ± 0.01	0.47 ± 0.02
LSD (*p* < 0.05)		0.22	0.03	0.20	0.23	0.02	0.02
*F*-value		20.43**	8.90**	2.38*	2.91**	17.77**	19.26**

Data are presented as the mean ± SD (n = 3); LSD, least significant difference; NS, non-significant; **significant at p < 0.01.

### 3.5 Correlation analysis

**Fig. 3:**
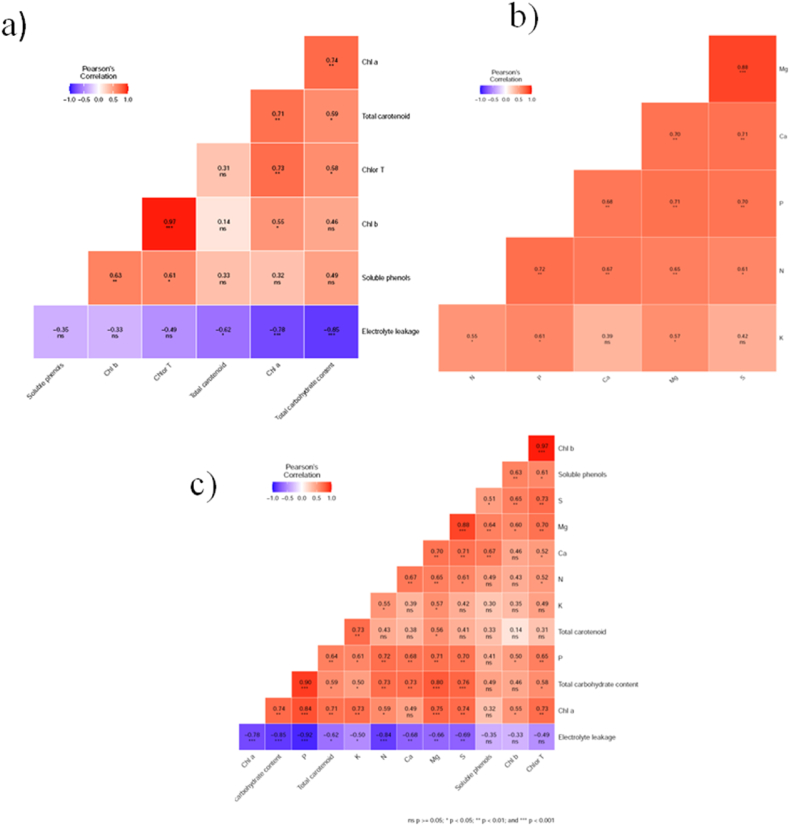
Correlation analysis a) between biochemical parameters, b) between plant nutrient concentration and c) between biochemical parameters combined with plant nutrient concentration. (* indicates statistically significant correlation at *P* ≤ 0.05, ** indicates statistically significant correlation at *P* ≤ 0.01 and *** indicates statistically significant correlation at *P* ≤ 0.001).

Correlation analysis between biochemical parameters revealed a statistically significant correlation at *P* ≤ 0.05 among total carotenoid-total carbohydrate content, total chlorophyll-total carbohydrate content, chlorophyll b-chlorophyll a and soluble phenol-total chlorophyll (Fig. 3a**)**. A significant correlation at *P* ≤ 0.01 was found among chlorophyll *a*-total carbohydrate content, total carotenoid-chlorophyll *a*, total chlorophyll-chlorophyll *a* and soluble phenol-chlorophyll *b* (Fig. 3a**)**. A highly significant correlation at *P* ≤ 0.001 was found only among chlorophyll *b*-total chlorophyll (Fig. 3a**)**. A significant negative correlation was found among electrolyte leakage-total carotenoid (*P* ≤ 0.05), electrolyte leakage-chlorophyll *a* (*P* ≤ 0.001), electrolyte leakage-total carbohydrate content (*P* ≤ 0.001) (Fig. 3a**)**.

In the case of plant nutrient concentration, a statistically significant correlation at *P* ≤ 0.05 was found among N–S, N–K, P–K and Mg–K (Fig. 3b**)**, whereas, a significant correlation at *P* ≤ 0.01 was found among Ca–Mg, Ca–S, P–Ca, P–Mg, P–S, N–P, N–Ca and N–Mg (Fig. 3b**)**. A highly significant correlation at *P* ≤ 0.001 was found only among Mg–S (Fig. 3b**)**.

Correlation analysis between biochemical parameters combined with plant nutrient concentration showed a highly significant correlation at *P* ≤ 0.001 between total carbohydrate content-S, total carbohydrate content-Mg, total carbohydrate content-P, chlorophyll a-Mg and chlorophyll a-P (Fig. 3c**)**. Interestingly, all the parameters showed a negative correlation with electrolyte leakage (Fig. 3c**)**.

### 3.6 Principal component analysis (PCA)

**Fig. 4:**
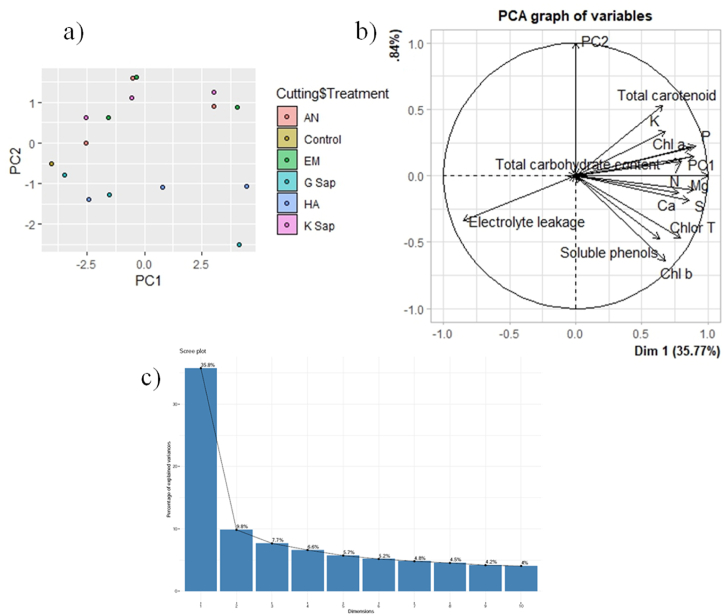
Principal component analysis (PCA) of biochemical parameters combined with plant nutrient concentration as influenced by various treatments (1, 5 and 10% solutions of G Sap, K Sap, AN, EM, HA and control) a) scatter plot, b) factor map and clustering of variables c) Scree plot.

PCA revealed that the first two principal components (PC1 and PC2) explained 75% of the total variation. While, the PC1 accounted for 63% of the total variation, the PC2 accounted for 11% of the total variation **(**Table 3**)**. Within PC1, chlorophyll *a*, chlorophyll *b*, total chlorophyll, total carotenoid, total carbohydrate content, soluble phenols, nitrogen, phosphorus, potassium, calcium, magnesium and sulphur showed positive loadings, while only electrolyte leakage showed negative loadings (Fig. 4 and Table 3). In PC2, only chlorophyll *a*, total carotenoid, total carbohydrate content, nitrogen, phosphorus and potassium showed positive loading and the rest of the variables revealed negative loadings (Fig. 4 and Table 3). The factor map (component plot) and clustering of all the variables (Fig. 4a) which revealed the distance between the variables and the origin and assessed the quality of the variables, were both depicted. Factor map and clustering of variables and scree plot were also presented in Fig. 4 b and c. Positively associated variables are clustered together, whereas variables with a negative correlation are placed on opposite sides of the plot's origin.

**Table 3:** Factor loadings of biochemical and nutrient parameters along with percentage of variance and cumulative variance accounted for each component.

Trait	PC1	PC2
Chl a	0.30	0.17
Chl b	0.23	−0.51
Chlor T	0.28	−0.38
Total carotenoid	0.23	0.42
Total carbohydrate content	0.31	0.12
Electrolyte leakage	−0.30	−0.27
Soluble phenols	0.22	−0.38
N	0.28	0.09
P	0.31	0.18
K	0.24	0.27
Ca	0.27	−0.10
Mg	0.31	−0.09
S	0.30	−0.15
*PV*	0.63	0.11
*CV*	0.63	0.75

PC, principal component; Chl a, chlorophyll a; Chl b, chlorophyll b; Chlor T, total chlorophyll; N, nitrogen; P, phosphorus; K, potassium; Ca, calcium; Mg, magnesium; S, sulphur; PV, percentage variance; CV, cumulative variance.

### 3.7 Gene expression

**Fig. 5:**
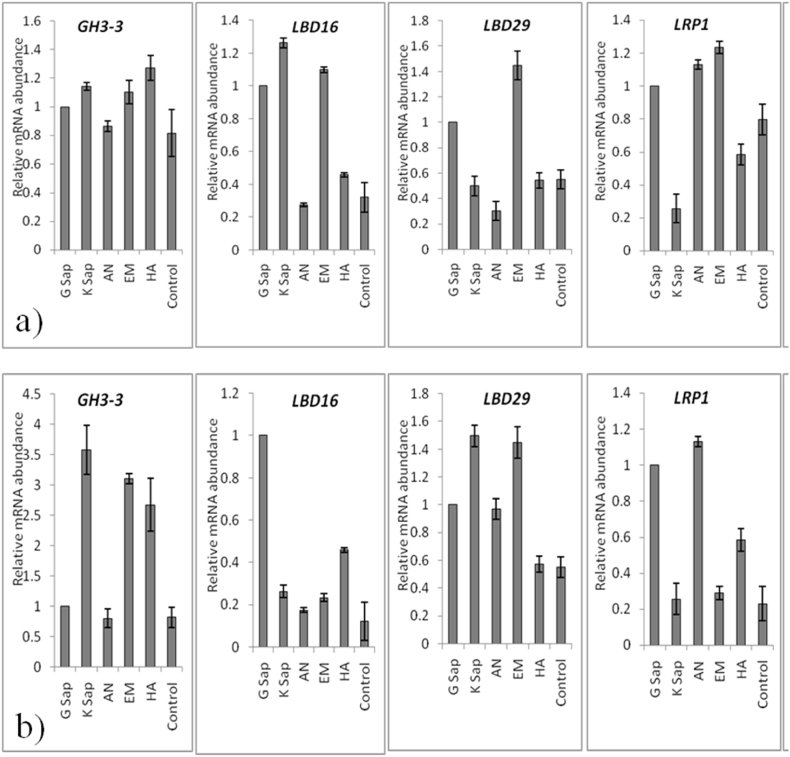
Relative expressions of root inducing genes (*GH3-3, LBD16, LBD29* and *LRP1*) in a) leaves and b) roots of cultivar ‘Hayward’ by qRT-PCR after treatment with G Sap, K Sap, AN, EM, HA @ 10% and control. Data are means ± SeM of n = 3 biological replicates.

With 18S-rRNA serving as the internal housekeeping gene, four candidate genes (*GH3-3, LBD16, LBD29* and *LRP1*) were chosen for qPCR analysis to validate the expression of these genes in leaves (Fig. 5a) and roots (Fig. 5b) of cultivar ‘Hayward’ by treatment of G Sap at 10%, K Sap at 10%, AN at 10%, EM at 10%, HA at 10% and control (Fig. 5). Based on their significance in plant adventitious root development, these rooting-related genes were chosen [41]. Generally, the leaves and roots of kiwifruit exhibited higher expression of all five genes with the application of all the treatments as compared to the control. In leaves, the gene *GH3-3* showed maximum expression with HA at 10%, the gene *LBD16* showed maximum expression with K Sap at 10%, the gene *LBD29* showed maximum expression with EM at 10% and the gene *LRP1* showed maximum expression with EM at 10%. While, in roots, the gene *GH3-3* showed maximum expression with HA at10%, the gene *LBD16* showed maximum expression with G Sap at 10%, the gene *LBD29* showed maximum expression with K Sap at 10% and the gene *LRP1* showed maximum expression with AN at 10%.

## 4 Discussion

Seaweed extracts possess a large number of bioactive compounds and a wide spectrum of valuable products are made out of them that are commercialized in the pharmaceutical, nutraceutical, cosmetic, food, feed, fertilisers and energy industries [23]. They are widely used for their multifarious benefits to crop growth and development, to prevent pests and diseases, and to improve the quality of the product and this has been proven time and again in a number of agri-horticultural crops [23,27,[45], [46], [47]]. Beside these, seaweed extracts have also been postulated to enhance rooting of cuttings in only a few species, like *Passiflora actinia* Hook [48], *Pinus patula* [49], *Lantana camara* and *Abelia* × *grandiflora* [50] and hybrid tea rose [51] etc. Despite these encouraging characteristics, in our experience, only a small number of seaweed extracts have been studied so far for root promotion in cutting propagation. Therefore, there is a good opportunity for using seaweed extracts for the promotion of rooting, the survival of cuttings and consequently, better vegetative and reproductive growth. As seaweed contains a lot of Iodine, therefore its effects on the cuttings like accumulation or any other biochemical or physiological changes, may be studied in the future.

In our experiment, various treatments (1, 5, 10 and 50% solutions of G Sap, K Sap, AN, EM, HA and control) were applied for six months at a 15 day interval to see the effect on the rooting of Kiwifruit cultivars *viz*. ‘Monty’, ‘Abott’, ‘Hayward’, ‘Allison’ and ‘Bruno’. Rooting is the first indication of the survival of a cutting, higher rooting percentage means more survival and consequently, success. All the treatments exhibited a significant effect on the rooting percent in all the kiwifruit cultivars (*P* ≤ 0.01). Interestingly, it was observed that EM at 5% and 10% gave the highest rooting in four kiwifruit cultivars. This can be attributed to the presence of rooting factors in a seaweed concentrate prepared from *Ecklonia maxima* which was investigated way back 1991, when evidence for the existence of heat stable, translocatable root promoters in seaweed concentrate made from the brown alga *Ecklonia maxima* (Osbeck) Papenfuss was discovered using the mung bean rooting bioassay [52]. Moreover, in *Ecklonia maxima* (Osbeck) Papenfuss numerous auxins (indole-3-acetic acid, four amino acid conjugates, and three more conjugates), cytokinins (free bases, O-glucoside derivatives, and aromatic cytokinins) and the ethylene precursor 1-aminocyclopropane-1-carboxylic acid are present, which have a crucial role in root growth and development [53,54]. It is yet not known how to increase the phytohormone concentration in the seaweed saps (like by artificially growing or naturally increasing in their habitat). There are questions about their availability in the market for users; therefore, they need to be studied extensively across the coastal areas of continents.

Shoot growth parameters like leaf number, number of branches, plant height and shoot diameter of kiwifruits in our experiments have been found to be significantly improved with the application of seaweed sap. Shoot growth parameters are an indication of the survival of a cutting, higher shoot growth parameters mean more survival and consequently, success. In most of the cultivars, AN at 10% had a significantly higher effect on leaf number and number of branches, whereas, G Sap at 10% had a significantly higher effect on plant height and shoot diameter than other treatments. It appears that AN and G Sap are good sources of auxins and cytokinin which have a probable significant higher effect on kiwifruit shoot growth parameters [[55], [56], [57]]. Additionally, in one study, it was demonstrated that components of commercial *A. nodosum* extracts influence the quantity and localization of auxins, which may account, at least in part, for the improved plant growth in Arabidopsis seedlings with a DR5:GUS reporter gene construct [58]. Similar results have been reported with *Gracilaria edulis* sap in potato [59], in hybrid maize [60], in foxtail millet [61], with *Ascophyllum nodosum* in maize [62], in *Vigna aconitifolia* (Jacq.) Marechal [63] and in winter wheat [64] and many more.

In our present work, root growth parameters have also been shown to be improved with the application of seaweed sap. Root growth parameters are an indication of the survival of a cutting, higher root growth parameters mean more survival and consequently, success. While, the application of AN at 5 and 10% improved the number of roots per cutting and K Sap and AN at 10% improved root length, HA at 10% improved root diameter and AN at 10% improved root weight. It appears that, the positive effect of seaweed sap in general and AN and K Sap in particular may be attributed to factors such as increased nutrient availability and uptake by the roots [65], regulation of auxin-responsive promoters [58], prevention of injury of the root tip and its meristematic tissues from stresses [66], attraction of beneficial microbes through root exudation [67], presence of palmitic acid (fatty acid) [68] etc. Similar observations of an increase in root growth parameters have been observed in pepper [69], lettuce [68], foxtail millet [61], papaya and passionfruit [70].

For the sake of convenience, we selected ‘Hayward’ cultivar for biochemical analysis. In general, pigments like chlorophyll *a*, chlorophyll *b*, total chlorophyll and total carotenoid contents increased in the leaves of kiwifruit cuttings with the application of seaweed extracts (mostly at 10%). As it is evident that seaweeds are a potent source of betaines (Glycinebetaine, ℽ-aminobutyric acid betaine and δ-aminovaleric acid betaine), it can be assumed that increased chlorophyll contents were recorded in the leaves of kiwifruit cuttings, similar to the results in tomato and cucumber cotyledons [71]. In the cucumber cotyledon bioassay, at dilutions between 0.2% and 3.5%, the seaweed extract produces “peaks” of activity that span the concentration range of 1 to 10^−2^ mg 1^−1^ of the various betaines. In our experiment, the total carbohydrate content was found to increase with the application of seaweed sap, which was highest with EM at 10%. The possible reason for this effect might be due to the increased chlorophyll content, which in turn enhanced photosynthesis and increased carbohydrate concentration. Similar results were revealed in alternate-bearing apple trees [72] and tomato plants [73]. Electrolyte leakage is an indicator of plant stress and it was found to be less in seaweed sap treated cuttings as compared to non-treated cuttings. This effect can be attributed to the presence of leakage inhibitors like abscisic acid, which has also been found in pepper seeds and egg plants treated with seaweed extracts, which gave positive results [27,74,75]. Soluble phenols were also found to be in significantly higher quantities in seaweed extract applied cuttings, which might be because of the activation of the antioxidant defence system and antioxidant enzymes to defend against damaging environmental factors [75].

Our experiment showed that, seaweed extract application led to significantly higher macro- and micronutrient accumulations in the kiwifruit cuttings as compared to the control plants. Treatment with fertiliser had a favourable impact on root growth metrics such as root dry weight, root volume, root length, and root length density when compared to an unfertilized plot during the entire development cycle in the case of wheat [76]. These findings are supported by one previous study [77], which showed enhanced absorption of magnesium, potassium, and calcium in lettuce treated with seaweed concentrates and another study that showed enhanced absorption of N, P, K and Mg in grapes and cucumber [78,79]. The increased stomatal efficiency in treated plants [78] and greater root development and antioxidant defence systems, may contribute to improved plant nutrient uptake [80,81].

Correlations between different nutrient elements and biochemical constituents helped in assessing the association among them in the samples [82,83]. The correlation among pigments, nutrient elements and biochemical constituents of seaweed sap gave us a good perception of how they are interrelated and their degree of association [84]. Application of seaweed saps like K sap recorded significant enhancement of carotenoids and total chlorophyll concentration in three durum wheat varieties in both the vegetative and reproductive stages [85] and decreased the leakage of electrolytes. A study on seven seaweeds and their saps showed a high degree of correlation (*R*^2^ = 0.992) [48]. The patterns of correlation for different nutrients, viz., N, P, K, Ca, Mg and S showed high variability among them in our study. Apart from reflecting statistical abundance, correlation among particular nutrient elements also has biological significance in plant growth and development [86]. There was a significant positive correlation among nutrients like N, P, K and S [87] which helped in the optimum balance of these elements in plants which may be required for optimum plant growth and development [88]. Magnesium (Mg) is a major abundant nutrient element in plant cells [89] and has an important role with a very close association with other nutrients like N, P and S [82]. A correlation at the highest level (p> 99.9%) was found among Mg and S in the experiment. The uptake of Mg^2+^ in roots is recorded to be inhibited if there are more than 20 μmol L^−1^ concentrations of K^+^ [89] and that’s why the correlation between Mg and K may not be significant at higher levels of probability (p > 99.9%) but significant at lower levels of probability (p > 95%) [90]. The presence of S in soil or its application through seaweed saps or other medium is known to enhance the Mg level in plant tissue due to their close association [91] and that’s why their (Mg–S) correlation was found to be highly significant (*P* ≤ 0.001). The concentrations of carotenoids and chlorophyll are negatively correlated with electrolyte leakage which may lead to membrane damage [92].

Understanding the link between variables can be aided by multivariate statistical analysis such as PCA. These could be useful in figuring out the nature of defining attributes and simplifying the data collection process. PCA analysis confirmed our findings (Table 3 and Fig. 4) with strong and positive correlations among chlorophyll *a*, chlorophyll *b*, total chlorophyll, total carotenoid, total carbohydrate content, soluble phenols, nitrogen, phosphorus, potassium, calcium, magnesium and sulphur in PC 1. Whereas, there are strong and positive correlations among chlorophyll *a*, total carotenoid, total carbohydrate content, nitrogen, phosphorus and potassium in PC 2.

In our experiment, four candidate genes (*GH3-3, LBD16, LBD29* and *LRP1*) were chosen for qPCR analysis to validate the expression of these genes in leaves and roots of cultivar ‘Hayward’ by treatment with G Sap at 10%, K Sap at 10%, AN at 10%, EM at 10%, HA at 10% and control (Fig. 5). As there is very limited information on the adventitious root formation genes, based on their significance, we have chosen four candidate genes (*GH3-3, LBD16, LBD29* and *LRP1*) in plant adventitious root development [41]. Our results suggested that leaves and roots exhibited higher expression of four genes with the application of all the treatments as compared to the control. Mainly, seaweed extract at 10% showed higher expression of all the genes in the root as well as the leaf tissues. GH3-3 protein is involved in auxin-conjugating enzyme, *LATERAL ORGAN BOUNDARIES-DOMAIN16* (*LBDI6*) and *LBD29* are involved in lateral root formation in rice, *LATERAL ROOT PRIMORDIUM1* (*LRP1*) is involved in auxin functioning [[93], [94], [95]]. The acquisition of pluripotency in callus cells is mediated by the transcription factor gene LATERAL ORGAN BOUNDARIES DOMAIN16 (*LBD16*) of *Arabidopsis thaliana*. The freshly formed callus on the callus-inducing medium expresses *LBD16* specifically, which is triggered by WUSCHEL RELATED HOMEOBOX11 (*WOX11*), and its expression immediately declines when the callus is transferred to the shoot-inducing medium [96]. It appears that, transcript levels of *GH3-3, LBD16, LBD29* and *LRP1* are upregulated in response to the seaweed extracts, which contain considerable quantities of auxin in them [97]; in previous research these genes have been found to be upregulated by auxin treatments or in relation to auxin [[93], [94], [95]]. So, it is clear that there is a direct relationship between auxin containing seaweed extracts and adventitious root formation in kiwifruit. Therefore, future research must focus on efforts to increase the auxin content in seaweed species through breeding approaches, so that they can be used as a rooting promoter in woody species like kiwifruit. Moreover, other root promoting genes that are involved in phytohormone biosynthesis pathways, nutrient accumulation pathways or other metabolic pathways need to be studied to come to an exact conclusion regarding the role of seaweed extract in promoting the rooting of cuttings.

## 5 Conclusion

In several agricultural and horticultural crops, seaweed extracts have demonstrated remarkably beneficial effects on crop growth, quality, reproduction and yield. Seaweed extracts can be used as an alternative to artificial rooting stimulants to aid in the rooting and development of cuttings in perennial fruit species like kiwifruit (*Actinidia deliciosa*) and thus might be used in organic farming. All of the treatments had a statistically significant impact on the percentage of kiwifruit that rooted, including the cultivars “Monty,” “Abott,” “Hayward,” “Allison,” and “Bruno”. The application of seaweed extracts resulted in a favourable increase in all shoot and root growth indices, including leaf number per cutting, number of roots per cutting, number of branches, plant height, shoot diameter, root length, root diameter, and root weight. In comparison to controls, cuttings treated with seaweed extract showed greater expression of all four root-promoting candidate genes (GH3-3, LBD16, LBD29, and LRP1). As a result, it can be concluded that seaweed extract and humic acid are excellent substitutes for synthetic hormones for encouraging rooting and growth in kiwifruit cuttings.

## Author contribution statement

Sudip Kumar Dutta: Conceived and designed the experiments, Wrote the paper.

Jayanta Layek: Contributed reagents, materials, analysis tools or data.

Ashish Yadav; Ramgopal Laha; Heiplanmi Rymbai; Somnath Mandal; Nandita Sahana: Analyzed and interpreted the data.

TL Bhutia; EL Devi: Performed the experiments.

VK Mishra; VB Patel: Analyzed and interpreted the data.

## Data availability statement

Data included in article/supplementary material/referenced in article.

## Additional information

Supplementary content related to this article has been published online at [URL].

## Declaration of competing interest

The authors declare that they have no known competing financial interests or personal relationships that could have appeared to influence the work reported in this paper.
