# Supplementary material for: Improvement of rooting and growth in kiwifruit (Actinidia deliciosa) cuttings with organic biostimulants
Source: Heliyon. 2023 Jun 30;9(7):e17815. doi: 10.1016/j.heliyon.2023.e17815 (PMC10339021; doi:10.1016/j.heliyon.2023.e17815)
Supplement: Multimedia component 1 [file mmc1.docx]

|  | **G Sap** | **K Sap** | **AN** | **EM** | **HA** |
| --- | --- | --- | --- | --- | --- |
| IAA | 8.67 (mg/L) | 21.11 (mg/L) | 0.05 (g/15mlH20) | Present | 14 to 18 ppm |
| ABA |  |  | 0.02 (g/15mlH20) | 0.31 to 20.70 ( pg mL−1) |  |
| Kinetin | - | 9.21 (mg/L) |  | Present |  |
| Zeatin | 3.13 (mg/L) | 19.81 (mg/L) |  |  |  |
| GAs | ND | 25.72 (mg/L) |  | 184-596 (pg mL−1) |  |
| Brassinosteroids brassinolide (BL) |  |  |  | 384.72 to 793.23 (pg mL−1) | 10400 ppm |
| K^+^ | 682.1 (mg/L) | 33654 (mg/L) | 2–3% |  | 14600 ppm |
| Zn^2+^ | 0.628 (mg/L) | 4.7 (mg/L) | 50–200 (mg kg−1) |  | 3200 ppm |
| Mn^2+^ | 32.9 (mg/L) | 2.1 (mg/L) |  |  | 200 ppm |
| Fe^2+^ | 12.7 (mg/L) | 86.1 (mg/L) | 150–1000 (mg kg−1) |  | 4800 ppm |
| Cu^2+^ | 0.044 (mg/L) | 0.65 (mg/L) |  |  | 30 ppm |
| P^3+^ | ND | 17.40 (mg/L) | 0.1-1.15 % |  |  |
|  |  |  |  |  |  |
| References | [1] | [1] | [2,3] | [4,5] | [6–8] |

Supplemental Table A: Chemical composition of G Sap (*Gracilaria edulis*), K Sap (*Kappaphycus alvarezii*), AN (*Ascophyllum nodosum*), EM (*Ecklonia maxima*), HA (Humic acid)

Note: ND: Not detected

Supplemental Table B. Sequences of the primers used in qRT-PCR analysis.

| **Gene** | **GenBank accession** | **Forward primer (5′–3′)** | **Reverse primer (5′–3′)** |
| --- | --- | --- | --- |
| *GH3-3* | AT2G23170 | CAAACCAATCCTCCAAATGAC | ACTTATCCGCAACCCGACT |
| *LBD16* | AT2G42430 | CCAACAACAGGTGGCT TTC | GGTACTTTCCGAGCTGTGTCTTA |
| *LBD29* | AT3G58190 | TCTCCAACAACAGGTTGTGA AT | AAGGAGCCTTAGTAGTGTCTCCA |
| *LRP1* | AT5G12330 | CAACTTCTAACACACCACCTCAA | ACTCATCATCCCCGTCCTC |
|  |  |  |  |

References:

[1] J. Layek, A. Das, G.I. Ramkrushna, K. Trivedi, D. Yesuraj, M. Chandramohan, D. Kubavat, P.K. Agarwal, A. Ghosh, Seaweed sap: a sustainable way to improve productivity of maize in North-East India, Int. J. Environ. Stud. . 72 (2015) 305–315. https://doi.org/10.1080/00207233.2015.1010855.

[2] L. Pereira, L. Morrison, P.S. Shukla, A.T. Critchley, A concise review of the brown macroalga Ascophyllum nodosum (Linnaeus) Le Jolis, J. Appl. Phycol. 32 (2020) 3561–3584. https://doi.org/10.1007/S10811-020-02246-6/METRICS.

[3] A.R. Kingman, J. Moore, Isolation, Purification and Quantitation of Several Growth Regulating Substances in Ascophyllum nodosum (Phaeophyta), Bot. Mar. 25 (1982) 149–154. https://doi.org/10.1515/BOTM.1982.25.4.149/MACHINEREADABLECITATION/RIS.

[4] W.A. Stirk, D. Tarkowská, V. Turečová, M. Strnad, J. van Staden, Abscisic acid, gibberellins and brassinosteroids in Kelpak®, a commercial seaweed extract made from Ecklonia maxima, J. Appl. Phycol. 26 (2014) 561–567. https://doi.org/10.1007/S10811-013-0062-Z/METRICS.

[5] I.J. Crouch, M.T. Smith, J. van Staden, M.J. Lewis, G. V. Hoad, Identification of Auxins in a Commercial Seaweed Concentrate, J. Plant Physiol. 139 (1992) 590–594. https://doi.org/10.1016/S0176-1617(11)80375-5.

[6] Z. Ekin, Integrated use of humic acid and plant growth promoting rhizobacteria to ensure higher potato productivity in sustainable agriculture, Sustainability. 11 (2019) 3417.

[7] M.T. Rose, A.F. Patti, K.R. Little, A.L. Brown, W.R. Jackson, T.R. Cavagnaro, A meta-analysis and review of plant-growth response to humic substances: practical implications for agriculture, Adv. Agron. 124 (2014) 37–89.

[8] K. Jindo, S.A. Martim, E.C. Navarro, F. Pérez-Alfocea, T. Hernandez, C. Garcia, N.O. Aguiar, L.P. Canellas, Root growth promotion by humic acids from composted and non-composted urban organic wastes, Plant Soil. 353 (2012) 209–220.
